# AKT3 promotes prostate cancer proliferation cells through regulation of Akt, B-Raf & TSC1/TSC2

**DOI:** 10.18632/oncotarget.4553

**Published:** 2015-07-10

**Authors:** Hui-Ping Lin, Ching-Yu Lin, Chieh Huo, Yee-Jee Jan, Jen-Chih Tseng, Shih Sheng Jiang, Ying-Yu Kuo, Shyh-Chang Chen, Chih-Ting Wang, Tzu-Min Chan, Jun-Yang Liou, John Wang, Wun-Shaing Wayne Chang, Chung-Ho Chang, Hsing-Jien Kung, Chih-Pin Chuu

**Affiliations:** ^1^ National Institute of Cancer Research, National Health Research Institutes, Miaoli County, Taiwan; ^2^ Institute of Cellular and System Medicine, National Health Research Institutes, Miaoli County, Taiwan; ^3^ Department of Life Sciences, National Central University, Taiwan; ^4^ Department of Pathology and Laboratory Medicine, Taichung Veterans General Hospital, Taichung City, Taiwan; ^5^ Medical College of Chung Shan Medical University, Taichung City, Taiwan; ^6^ Institute of Molecular and Cellular Biology, National Tsing Hua University, Hsinchu, Taiwan; ^7^ Department of Medical Education and Research, China Medical University Beigan Hospital, Yunlin, Taiwan; ^8^ Institute of Molecular and Genomic Medicine, National Health Research Institutes, Miaoli County, Taiwan; ^9^ Graduate Institute of Basic Medical Science, China Medical University, Taichung City, Taiwan; ^10^ Graduate Program for Aging, China Medical University, Taichung City, Taiwan; ^11^ Ph.D. Program in Tissue Engineering and Regenerative Medicine, National Chung Hsing University, Taichung City, Taiwan; ^12^ Ph.D. program in Environmental and Occupational Medicine, Kaohsiung Medical University, Kaohsiung City, Taiwan

**Keywords:** Akt3, prostate cancer, B-Raf, TSC1/2, proliferation

## Abstract

The qRT-PCR analysis of 139 clinical samples and analysis of 150 on-line database clinical samples indicated that AKT3 mRNA expression level was elevated in primary prostate tumors. Immunohistochemical staining of 65 clinical samples revealed that AKT3 protein expression was higher in prostate tumors of stage I, II, III as compared to nearby normal tissues. Plasmid overexpression of AKT3 promoted cell proliferation of LNCaP, PC-3, DU-145, and CA-HPV-10 human prostate cancer (PCa) cells, while knockdown of AKT3 by siRNA reduced cell proliferation. Overexpression of AKT3 increased the protein expression of total AKT, phospho-AKT S473, phospho-AKT T308, B-Raf, c-Myc, Skp2, cyclin E, GSK3β, phospho-GSK3β S9, phospho-mTOR S2448, and phospho-p70S6K T421/S424, but decreased TSC1 (tuberous sclerosis 1) and TSC2 (tuberous Sclerosis Complex 2) proteins in PC-3 PCa cells. Overexpression of AKT3 also increased protein abundance of phospho-AKT S473, phospho-AKT T308, and B-Raf but decreased expression of TSC1 and TSC2 proteins in LNCaP, DU-145, and CA-HPV-10 PCa cells. Oncomine datasets analysis suggested that AKT3 mRNA level was positively correlated to BRAF. Knockdown of AKT3 in DU-145 cells with siRNA increased the sensitivity of DU-145 cells to B-Raf inhibitor treatment. Knockdown of TSC1 or TSC2 promoted the proliferation of PCa cells. Our observations implied that AKT3 may be a potential therapeutic target for PCa treatment.

## INTRODUCTION

Prostate cancer (PCa) is one of the most common non-cutaneous carcinoma of men in Western countries, especially in United States. The statistics of American Cancer Society estimated that 238, 590 new cases of PCa were diagnosed and approximately 29, 720 people died from PCa-specific deaths in United States in 2013. Incidence of PCa is increasing steadily in most countries. Phosphatase and tensin homolog (PTEN) protein is a phosphatase dephosphorylating phosphatidylinositol (3, 4, 5)-trisphosphate. PTEN is a negative regulator for phosphoinositide 3-kinase (PI3K)-AKT signaling pathway [1]. Deletion of PTEN was observed in 40–70% of PCA patients, resulting in upregulation of PI3K-AKT signaling. Deletion or mutation of PTEN is associated with poor prognosis, cancer metastasis, and progression towards castration-resistant status of prostate cancer [2–4]. PI3K-AKT signaling plays an important role in the survival of PCa cells [2–4]. Up-regulation of PI3K-AKT activity is associated with poor clinical outcome of PCa [2, 5–8]. AKT is a serine/threonine protein kinase regulating a variety of cellular responses. Three isoforms, AKT1, AKT2, and AKT3 exist in mammalian. There are two phosphorylation sites on all AKT isoforms, the threonine 308 and serine 473. These two phosphorylation sites regulate the activity of AKT [9, 10]. Level of phospho-AKT correlates with higher Gleason score [11]. The mRNA expression of AKT1, AKT2, and AKT3 are detected in both normal and prostate cancer tissues [12]. Transient knockdown of AKT1, AKT2, or AKT3 using siRNA suppresses the proliferation of prostate cancer cells both *in vitro* and *in vivo* [13, 14]. High level of phosphorylated AKT1 is a strong predictor for prostate cancer recurrence [5] while AKT2 is essential for survival of PTEN-deficient prostate tumors [15]. The molecular mechanisms how AKT1 and AKT2 regulate proliferation and survival or prostate cancer cells has been extensively studied. However, the clinical significance of AKT3 is not clear and how AKT3 may promote prostate cancer cell proliferation is not understood.

LNCaP, PC-3, and DU-145 are commonly used PCa cell lines. The LNCaP PCa cell line was established from a human lymph node metastatic lesion of prostatic adenocarcinoma. PC-3 and DU-145 cells were androgen receptor (AR)-negative PCa cells established from human prostatic adenocarcinoma metastatic to bone and brain, respectively [16–18]. The proliferation of LNCaP cells is androgen-dependent while the proliferation of PC-3 and DU-145 cells is androgen-insensitive. CA-HPV-10 is an AR-positive PCa cell line derived from a prostatic adenocarcinoma of Gleason Grade 4/4 transformed by transfection with HPV18 DNA9 [19]. In PC-3 and DU-145 cells, the abundance and enzymatic activity of AKT3 was approximately 20–60-fold higher than that in the LNCaP prostate cancer cells [14, 20, 21]. As PC-3 and DU-145 proliferate faster than LNCaP, we suspected that AKT3 may be involved in promotion of PCa cell proliferation. Additionally, we previously demonstrated that treatment with caffeic acid phenethyl ester (CAPE) suppresses proliferation of PC-3 cells dose-dependently via inhibiting AKT signaling [22]. We observed that under the treatment of CAPE in PC-3 and DU-145 cells, AKT3 is among the proteins whose abundance is decreased most by CAPE. The protein abundance of AKT3 decreased at least 4–5 folds more as compared to that of AKT1 and AKT2 in PC-3 and DU-145 cells treated with CAPE. We hypothesize that AKT3 may play important role in regulating prostate cancer cell proliferation. We therefore used the four PCa cell lines as well the online clinical datasets to investigate the molecular mechanisms how AKT3 promotes PCa cell proliferation.

## RESULTS

### Expression of AKT3 mRNA and protein level elevates in primary prostate tumors

**Figure 1:**
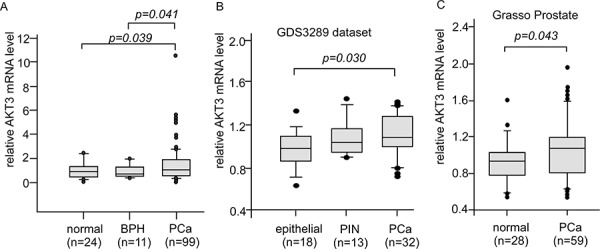
Expression levels of AKT3 mRNA in human normal, disease, and cancerous prostate tissues **A.** Box plots showed relative AKT3 mRNA level in 24 normal prostate tissues, 11 benign prostatic hyperplasia (BPH), and 99 primary tumors (stage I to III) from TissueScan Prostate Tissue qPCR Array HPRT501~503 assayed with qRT-PCR. The mRNA in each well was quantified to gene expression of β-actin. *p* value smaller than 0.05 was considered statistically significant. **B.** Box plots showed relative AKT3 mRNA level in 18 normal epithelial prostate tissues, 13 prostatic intraepithelial neoplasia (PIN), and 32 primary prostate tumors from PubMed GEO profile GDS3289 dataset. *p* value smaller than 0.05 was considered statistically significant. **C.** Box plots showed relative AKT3 mRNA level in 28 normal prostate gland tissues and 59 primary prostate tumors from Grasso Prostate dataset. *p* value smaller than 0.05 was considered statistically significant.

To determine the gene expression level of AKT3 in normal and cancerous prostate tissues, we assayed AKT3 mRNA level in 24 normal prostate tissue, 11 benign prostatic hyperplasia (BPH), and 99 primary tumors from TissueScan Prostate Tissue qPCR Array using quantitative real time-PCR (Figure 1A). Compared to normal prostate tissue and BPH, prostate primary tumors expressed higher AKT3 mRNA level (Figure 1A). Analysis of AKT3 mRNA expression level in 46 normal prostate epithelial tissues, 13 prostate intraepithelial neoplasia (PIN), and 91 primary prostate tumor from PubMed GEO profile dataset GSE6099 and Oncomine Grasso Prostate database also indicated that AKT3 mRNA expression in primary tumors was higher than that in normal prostate epithelial tissues (Figure 1B, 1C).

**Figure 2:**
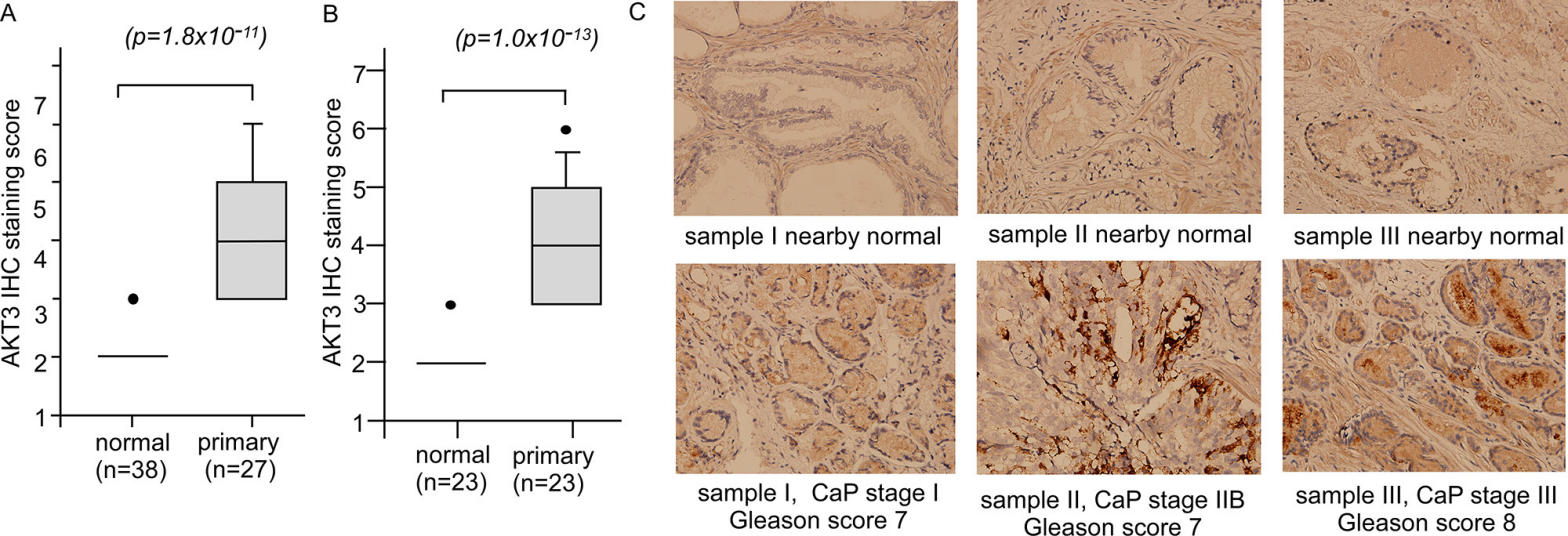
Protein expression of AKT3 in normal human prostate tissues versus prostate tumors **A.** Protein expression of AKT3 was assayed by IHC staining in 38 normal prostate epithelial tissues and 27 primary prostate tumors containing stage I to III. Among the tissues, 23 set of normal and cancerous prostate tissues are paired **B.** *p* value smaller than 0.05 was considered statistically significant. **C.** IHC staining of AKT3 in cancerous tissues of sample I (stage I, Gleason score 7), II (stage IIB, Gleason score 7), III (stage III, Gleason score 8), and their paired normal tissues was shown. The original magnification of IHC staining is 200X.

We further determined the protein level of AKT3 in normal and cancer prostate tissues by immunohistochemical staining (IHC) in 38 normal prostate epithelial tissues and 27 primary prostate tumors including stage I, II, and III (Figure 2A). Among the tissues, 23 of them are paired prostate tumor tissues and their nearby normal prostate tissues (Figure 2B). AKT3 protein expression was higher in primary prostate tumors as compared to normal tissue (Figure 2A, 2B). No significant difference in AKT3 protein expression level was observed among different stages (data not shown). Representative IHC staining images were shown in Figure 2C.

### Elevation of AKT3 protein level promotes proliferation of prostate cancer cells

**Figure 3:**
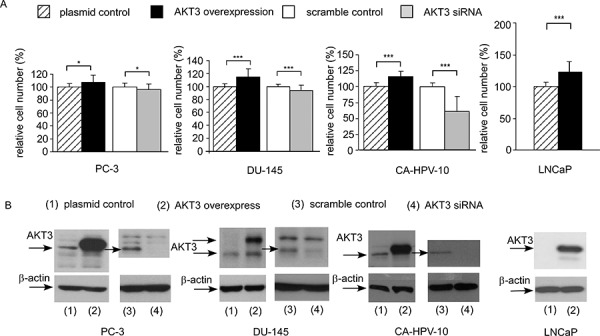
Overexpression and siRNA knockdown of AKT3 affected cell proliferation of PC-3, DU-145, CA-HPV-10, and LNCaP PCa cells **A.** Cellular proliferation of PC-3, DU-145, Ca-HPV-10, and LNCaP PCa cells with plasmid control, AKT3 overexpression, siRNA scramble control, or AKT3 siRNA knockdown was assayed by Hoechst dye-based 96-well proliferation assay 96 h after cell seeding Asterisk *and ***represents statistically significant difference *p* < 0.05 and *p* < 0.001, respectively, between the two group of cells being compared. **B.** Expression of AKT3 in PC-3, DU-145, Ca-HPV-10, and LNCaP PCa cells was confirmed by Western blotting. Protein abundance of β-actin was used as loading control.

**Figure 4:**
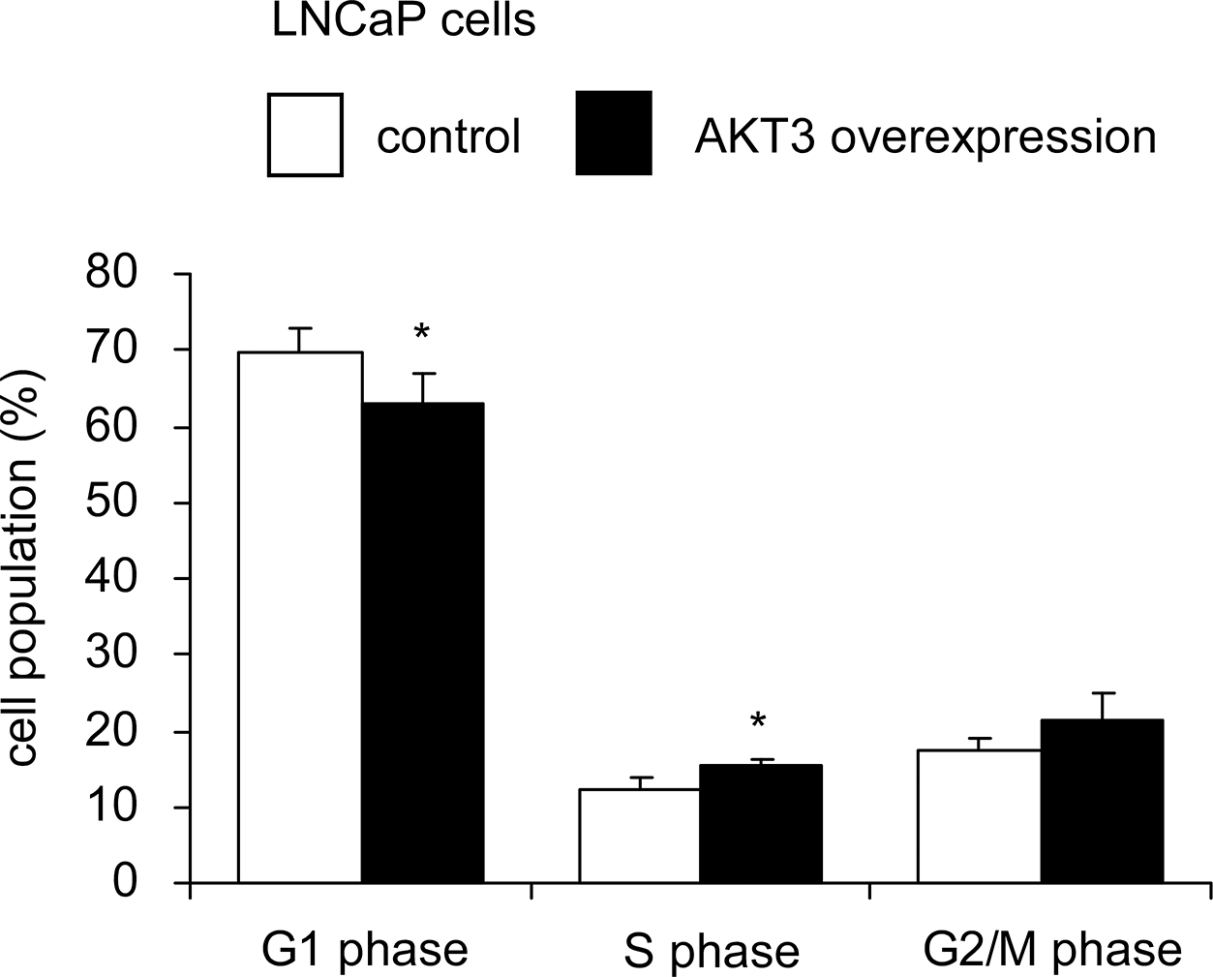
Overexpression of AKT3 promoted cell cycle progression in LNCaP cells Percentage of cell population of LNCaP cells with either plasmid control or AKT3 overexpression in G1 phase, S phase, and G2/M phase was analyzed by PI-staining flow cytometry. Values represent the mean +/− Standard Error derived from 5 independent experiments. Asterisk * denotes significant difference *p* < 0.05 between the AKT3 overexpressing cells as compared to the control cells.

**Figure 5:**
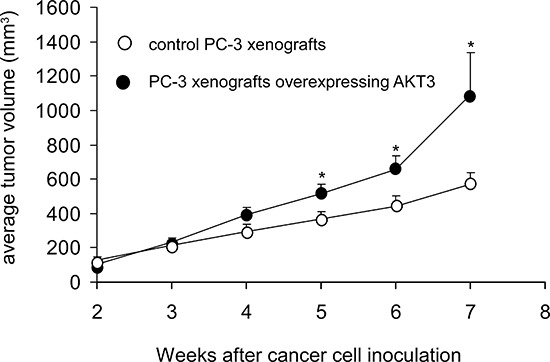
Overexpression of AKT3 stimulated tumor growth of PC3 xenografts in nude mice Male Balb/c nu/nu mice at age 6–8 weeks of age were injected subcutaneously in both flanks with 5 × 10^5^ PC-3 cells overexpressing either control vector or AKT3. After 2 weeks, the average tumor volume exceeded 100 mm^3^. The control group contained 7 mice and 11 tumors, while the AKT3 overexpression group contained 7 mice and 10 tumors. Tumor volume and body weight of mice carrying PC-3 xenografts was measured weekly using calipers and volume was calculated using the formula volume = length × width × height × 0.52 [31–34].

Overexpression of AKT3 increased 10–25% of cellular proliferation in PC-3, DU-145, CA-HPV-10, and LNCaP human prostate cancer cells (Figure 3A, 3B). Knockdown of AKT3 with siRNA decreased 5–30% of cellular proliferation in PC-3, DU-145, and CA-HPV-10 prostate cancer cells (Figure 3A, 3B). LNCaP cells do not express AKT3 (Figure 3B) and therefore we did not perform siRNA knockdown of AKT3 in LNCaP cells. The doubling time for control and AKT3 overexpressing LNCaP was 34.7 h and 32.5 h, respectively, as determined by proliferation assay. The 2.2 h difference in doubling time will result in approximately 15% increase of cell number in LNCaP overexpressing AKT3 cells after 96 h culture as compared to the control LNCaP cells, which is consistent to the observed result in Figure 3A. Overexpression of AKT3 decreased G1 phase population but increased S phase population in LNCaP cells (Figure 4). We injected PC-3 cells overexpressing control vector or AKT3 into nude mice (Figure 5). Elevation of AKT3 significantly promoted tumor growth of PC-3 xenografts in nude mice. The average volume of PC-3 tumor overexpressing AKT3 was twice the average volume of control PC-3 tumors (Figure 5). These results suggested that elevation of AKT3 protein promotes proliferation of human prostate cancer cells both *in vitro* and *in vivo*.

### Overexpression and siRNA knockdown of AKT3 protein expression affects the expression of signaling proteins in PCa cells

**Figure 6:**
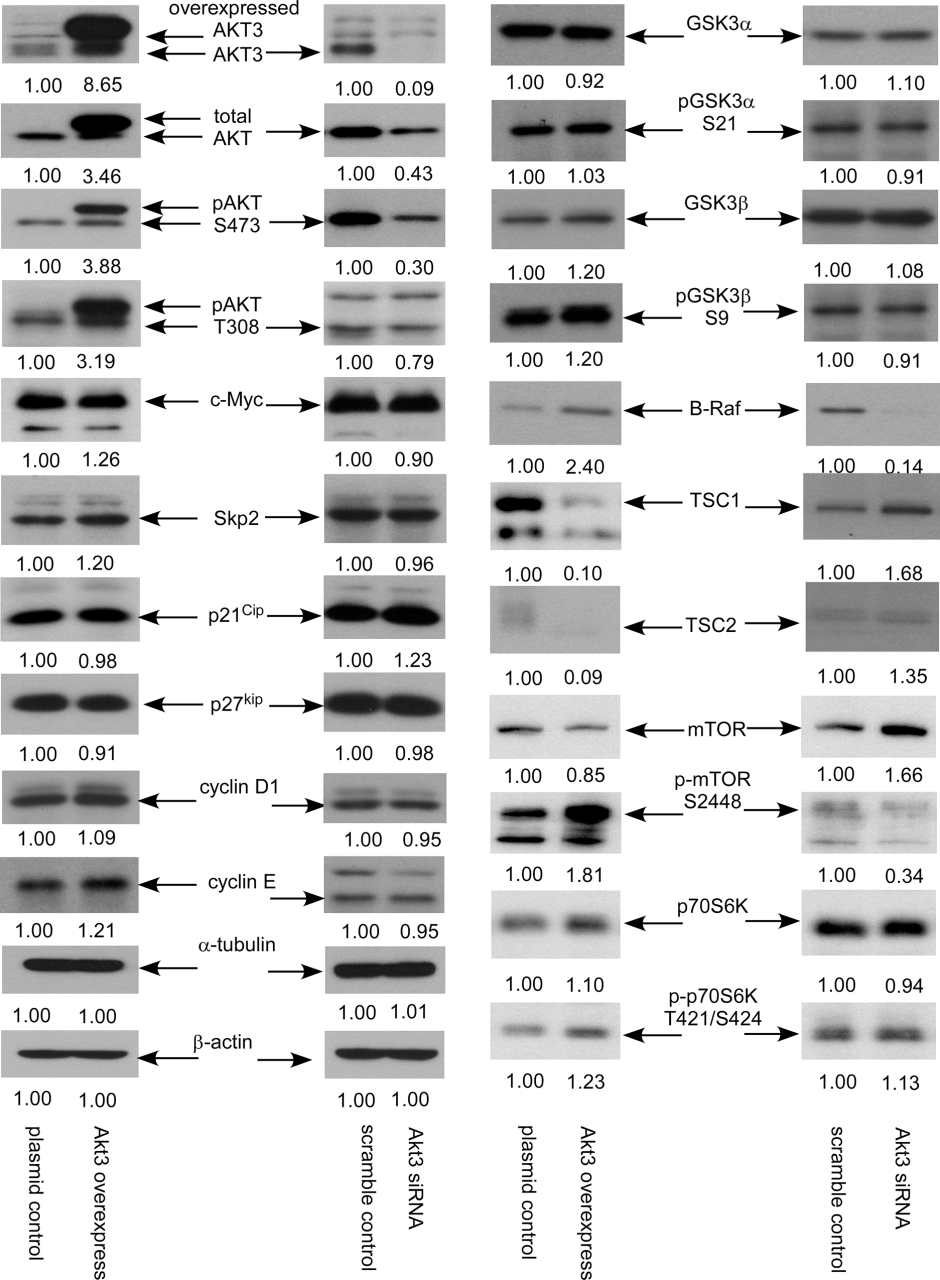
AKT3 overexpression and siRNA knockdown affected expression of signaling proteins in PC-3 cells Protein expression of total AKT, AKT3, phospho-AKT S473, phospho-AKT T308, c-Myc, Skp2, p21^Cip^, p27^Kip^, cyclin D1, cyclin E, GSK3α, phospho-GSK-3α S21, GSK3β, phospho-GSK-3β S9, B-Raf, TSC1, TSC2, mTOR, phosphor-mTOR S2448, p70S6K, phopsho-p70S6K T421/S424 in PC-3 vector control, PC-3 overexpressing AKT3, PC-3 scramble control, and PC-3 AKT3 siRNA knockdown was assayed by Western blotting. Protein abundance of α-tubulin and β-actin were used as loading control.

Overexpression of AKT3 caused at least 2-fold increase of protein expression of total AKT, phospho-AKT Ser473, phospho-AKT Thr308, and B-Raf, as well as increased the protein abundance of c-Myc, S-phase kinase-associated protein 2 (Skp2), cyclin E, GSK3β, phospho-GSK3β Ser9, phospho-mTOR Ser2448, and phospho-p70S6K Thr421/Ser424. Elevation of AKT3 also caused 90% reduction of TSC1 and TSC2 protein in PC-3 cells (Figure 6). On the other hand, knockdown of AKT3 with siRNA caused at least 50% reduction of total AKT, phospho-AKT Ser473, B-Raf, and phospho-mTOR Ser2448 as well as decreased phospho-AKT Thr308 in PC-3 cells. Reduction of AKT3 increased TSC1, TSC2, p21^Cip1^ and mTOR in PC-3 cells (Figure 6).

### Elevation of AKT3 protein increases protein abundance of B-Raf and phosphorylation of AKT while decreases expression of TSC1 and TSC2 proteins

**Figure 7:**
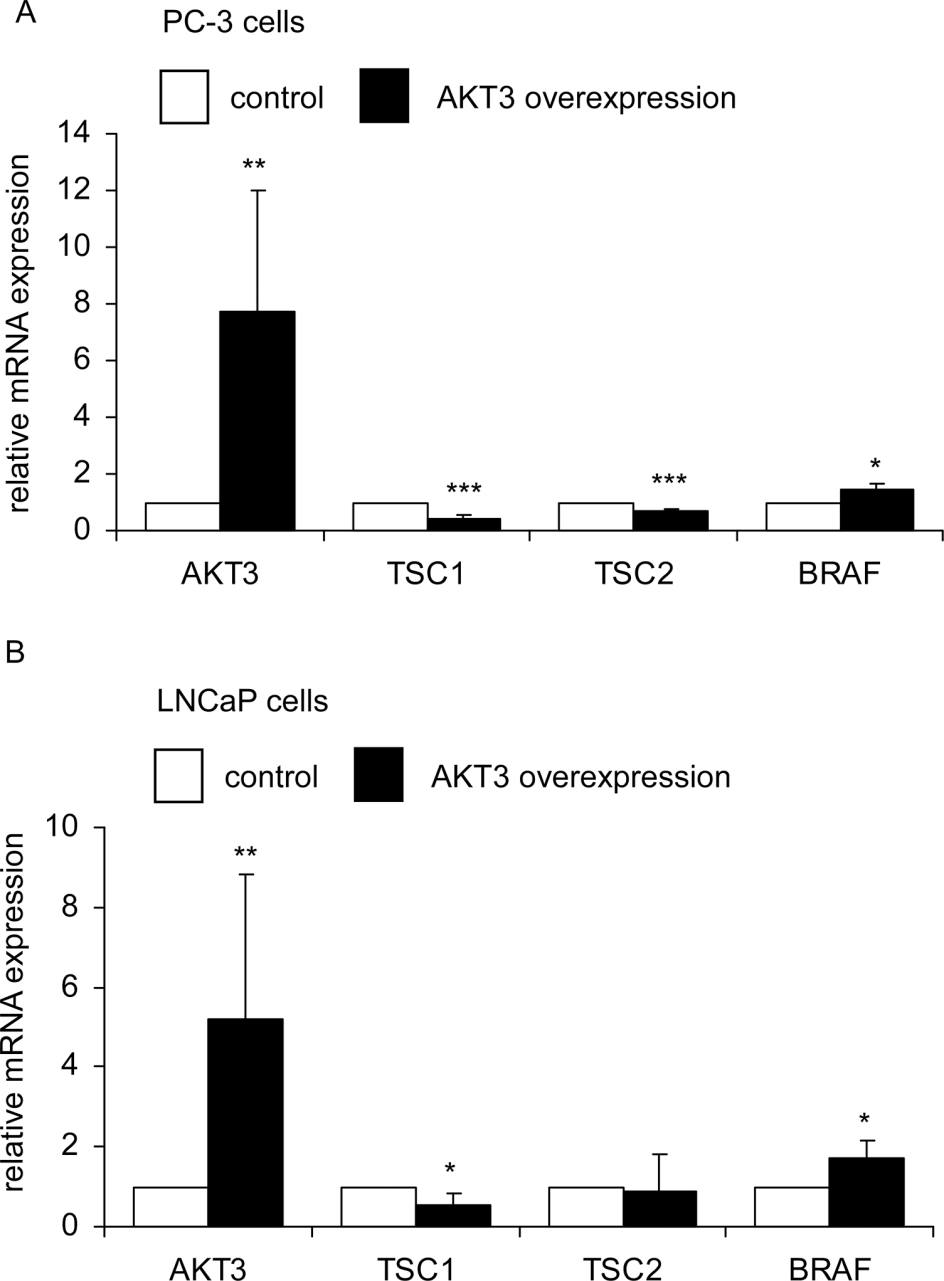
AKT3 gene overexpression affected expression of TSC1, TSC2, and BRAF genes in PC-3 and LNCaP PCa cells Expression of AKT3, TSC1, TSC2, and BRAF genes in PC-3 **A.** and LNCaP **B.** cells was determined by qRT-PCR. Asterisk*,**, and *** denotes significant difference *p* < 0.05, *p* < 0.01, and *p* < 0.001, respectively, between the AKT3 overexpressing cells as compared to the control cells. GAPDH were used as loading control.

**Figure 8:**
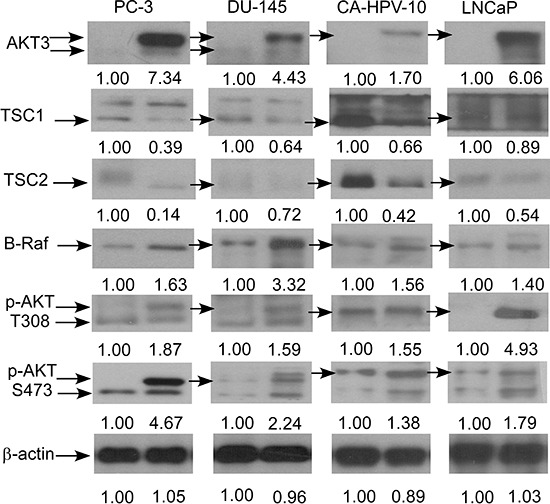
AKT3 overexpression affected protein expression of B-Raf, TSC1, TSC2, and AKT phosphorylation in PC-3, DU-145, CA-HPV-10, and LNCaP PCa cells Protein expression of AKT3, TSC1, TSC2, B-Raf, phospho-AKT S308, and phospho-AKT T473 in PC-3, DU-145, CA-HPV-10, and LNCaP cells overexpressing control vector or AKT3. Protein abundance of β-actin were used as loading control.

As cDNA overexpression of AKT3 and siRNA knockdown of AKT3 showed opposite effects on phospho-AKT S473, phospho-AKT T308, B-Raf, TSC1 and TSC2, these proteins may play essential roles in the proliferation regulation of AKT3 in prostate cancer cells. We therefore determined if overexpression of AKT3 also affects mRNA and protein expression of B-Raf, TSC1, and TSC2. Overexpression of AKT3 gene decreased mRNA expression of TSC1 in PC-3 and LNCaP cells, TSC2 in PC-3 cells, and increased BRAF gene expression in PC-3 and LNCaP cells (Figure 7A, 7B). Overexpression of AKT3 proteins decreased protein expression of TSC1 and TSC2 but increased protein abundance of phospho-AKT Ser473, phospho-AKT Thr308, and B-Raf in PC-3, DU-145, CA-HPV, and LNCaP PCa cells (Figure 8).

### Correlation between AKT3 and BRAF genes in prostate cancer tissues

**Figure 9:**
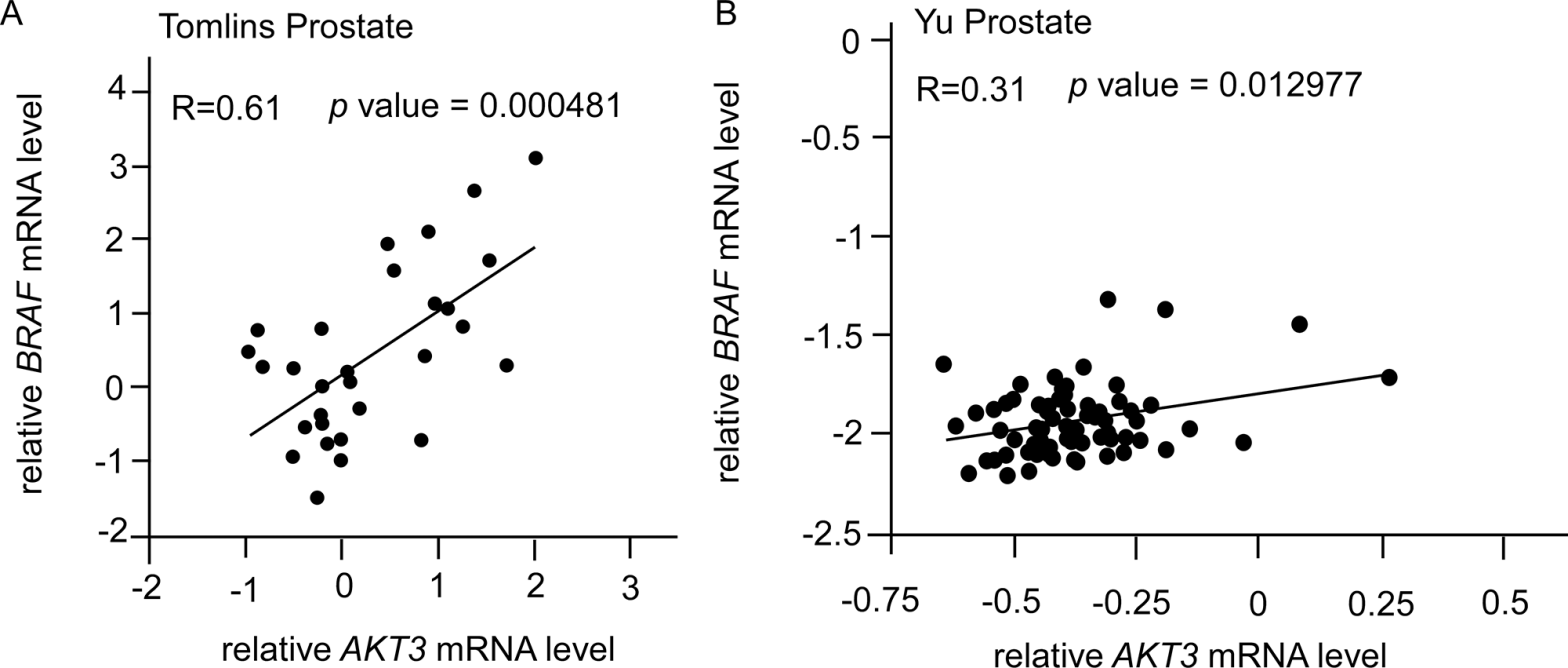
Correlation of gene expression levels of AKT3, BRAF, TSC1, and TSC2 in prostate cancer public domain database **A.** Scatter plots showing correlation of AKT3 and BRAF genes in Tomlins Prostate dataset (AKT3/IMAGE:360838; BRAF/IMAGE:417403; *R* = 0.61, *n* = 29) [28]. **B.** Scatter plots showing correlation of AKT3 and BRAF genes in Yu Prostate dataset (AKT3 probe: 40781_at; BRAF probe: 40306_at; *R* = 0.31, *n* = 64) [30]. The *r* value indicated correlation coefficient.

Based on the above observations *in vitro* that elevation of AKT3 increased protein level of B-Raf but decreased TSC1 and TSC2 protein abundance, we hypothesize that expression of BRAF gene will show positive correlation with AKT3, while expression of TSC1 and TSC2 will negatively correlate with AKT3. From online database, we observed that BRAF and AKT3 showed positive correlation (Figure 9A, 9B) with *p* value smaller than 0.05. Although the mRNA level of TSC1 and TSC2 genes showed a trend of negative correlation with AKT3, the *p* value was not statistically significant.

### LNCaP and DU-145 cells overexpressing AKT3 were more resistant to B-Raf inhibitor treatment while siRNA knockdown of AKT3 increased sensitivity of DU-145 cells to B-Raf inhibitor treatment

**Figure 10:**
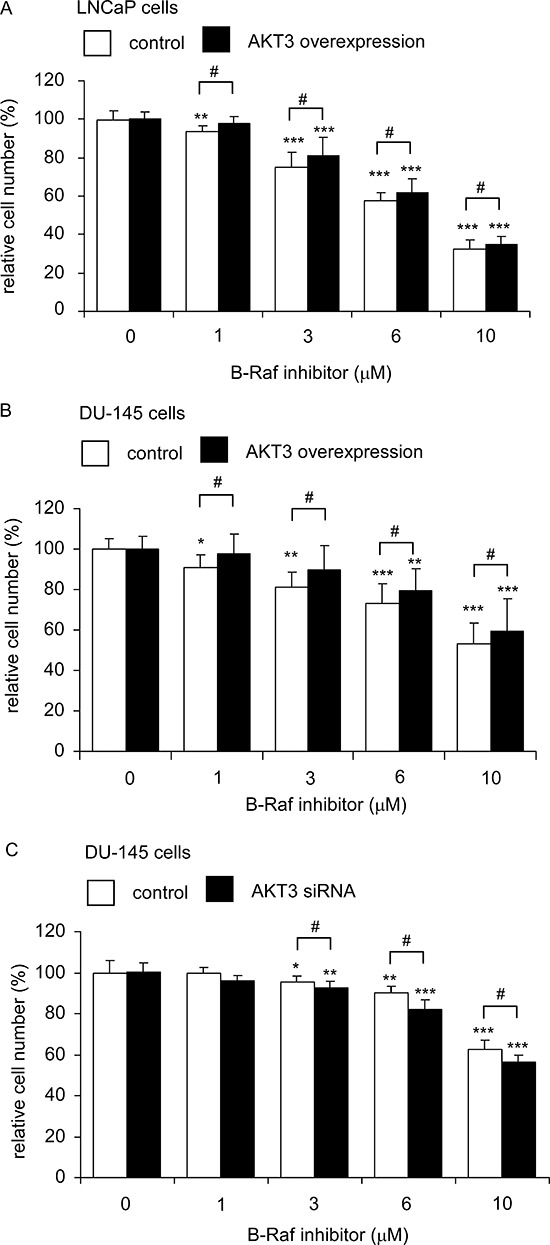
LNCaP and DU-145 cells overexpressing AKT3 was more resistant to B-Raf inhibitor treatment while siRNA knockdown of AKT3 increased sensitivity of DU-145 to B-Raf inhibitor treatment Cellular proliferation of LNCaP cells **A.** or DU-145 cells **B.** with plasmid control or AKT3 overexpression, or DU-145 cells **C.** with scramble control or AKT3 siRNA knockdown were being treated with increasing concentration of B-Raf inhibitor for 96 h was assayed by Hoechst dye-based 96-well proliferation assay. Asterisk *, ** and *** represents statistically significant difference *p* < 0.05, < 0.01 and *p* < 0.001, respectively, between B-Raf inhibitor treating cells and its own control group (no treatment). Number sign # represents statistically significant difference *p* < 0.05 between control cells and AKT3 overexpression (or AKT3 siRNA knockdown) cells.

Since overexpression of AKT3 induced protein expression of B-Raf, we predicted that PCa cells overexpressing AKT3 will be more resistant to treatment with B-Raf inhibitor. As shown in Figure 10, treatment with B-Raf inhibitor at 1–10 μM dose-dependently suppressed the proliferation of LNCaP and DU-145 cells. LNCaP cells (Figure 10A) and DU-145 cells (Figure 10B) overexpressing AKT3 were more resistant to B-Raf inhibitor treatment. On the other hand, siRNA knockdown of AKT3 in DU-145 cells increased the sensitivity of DU-145 cells to B-Raf inhibitor treatment (Figure 10C).

### Knockdown of TSC1 or TSC2 induces proliferation of prostate cancer cells

**Figure 11:**
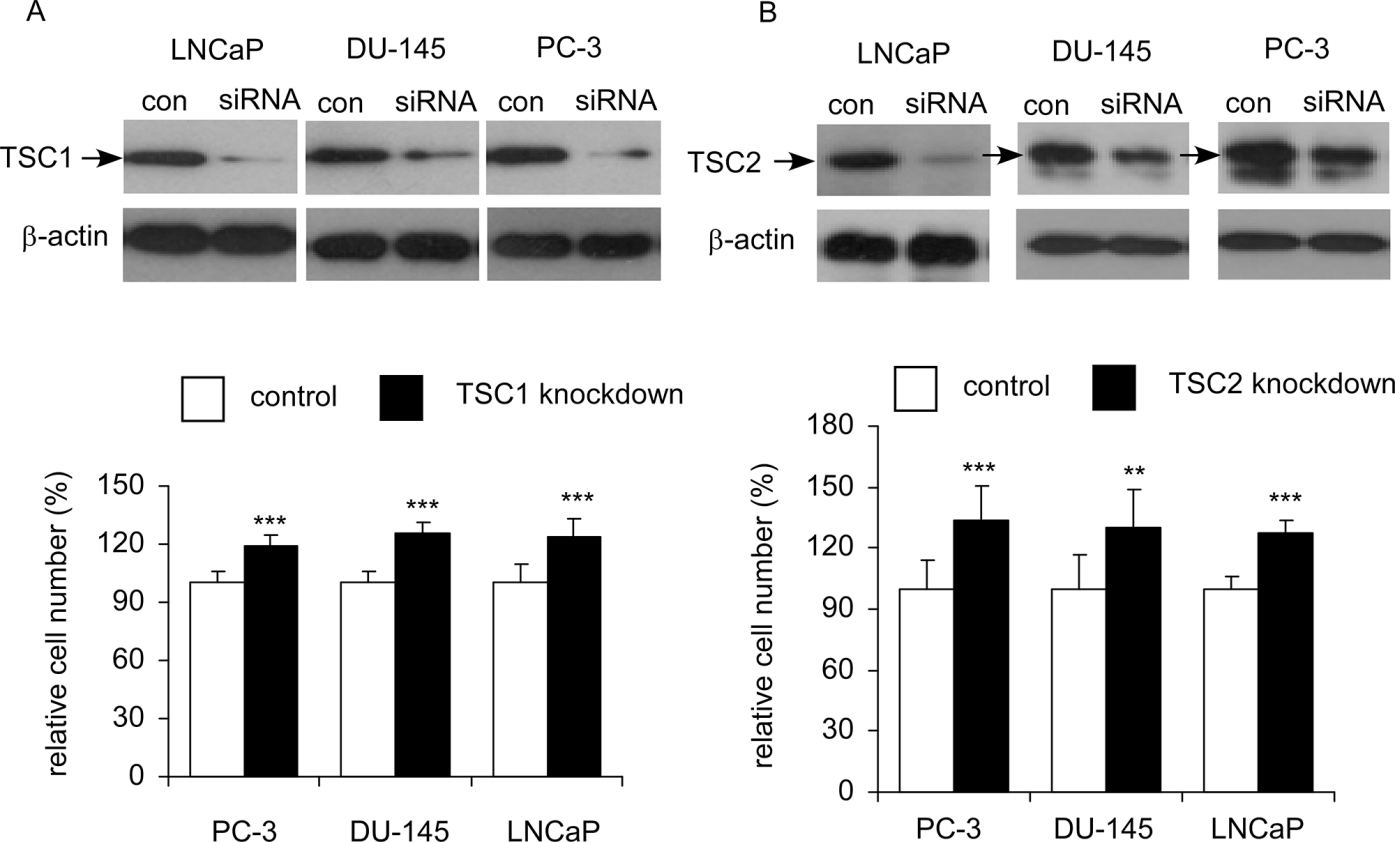
Effects of siRNA knockdown of TSC1 or TSC2 on cellular proliferation of LNCaP, PC-3, and DU-145 cells Cellular proliferation of LNCaP, PC-3, and DU-145 cells with siRNA knockdown of TSC1 **A.** or TSC2 **B.** was assayed by Hoechst dye-based 96-well proliferation assay after 96 h culture and was compared to the cells with scramble control. Asterisk ** and ***represents statistically significant difference *p* < 0.01 and *p* < 0.001, respectively, between siRNA knockdown and control. Western blotting assay was used to confirm the knockdown of TSC1 or TSC2 in these prostate cancer cells.

As overexpression of AKT3 decreased protein level of TSC1 and TSC2, we hypothesized that knockdown of TSC1 (Figure 11A) or TSC2 (Figure 11B) will enhance proliferation of prostate cancer cells. Indeed, siRNA knockdown of TSC1 or TSC2 in LNCaP, PC-3, or DU-145 cells significantly promoted cellular proliferation of these cells.

## DISCUSSION

To our knowledge, this is the first study examining how AKT3 promoting the proliferation of PCa cells and the clinical significance of AKT3 in PCa. We demonstrated that AKT3 mRNA and protein expression was elevated in primary prostate tumors as compared to normal prostate tissues. We noticed that the fold changes of AKT3 mRNA between normal vs. malignant prostate issues is small. However, protein is the actual players involved in most biological process. The IHC staining (Figure 2) indicated that AKT3 protein level is approximately two fold higher in primary prostate tumors as compared to nearby non-malignant prostate tissues. The significant elevation of AKT3 proteins in prostate tumors may allow the AKT3 to be a potential therapeutic target for prostate cancer. We discovered that the elevation of AKT3 promoted the proliferation of different prostate cancer cell lines via induction of AKT phosphorylation and B-Raf as well as the reduction of TSC1 and TSC2. Sasaki T et al., demonstrated that knockdown of AKT1, AKT2, or AKT3 by siRNA can suppress the proliferation of PC-3 and DU-145 prostate cancer cells [14], suggesting that all AKT isoforms are involved in the regulation of prostate cancer cells. In our study, although the overexpression of AKT3 in PC-3, DU-145, CA-HPV-10, and LNCaP (Figure 8) increased the phosphorylation of AKT on Ser473 and Thr308, the protein abundance of AKT1 and AKT2 was not changed (data not shown). Our observation suggested that AKT3 can regulate the proliferation of prostate cancer cells independent of AKT1 or AKT2.

Cyclin-D1 is a protein encoded by the CCND1 gene and is required for the cell cycle progression through G1 phase [35]. Complex between Cyclin D and Cdk4 or Cdk6 are key player for G1/S transition in cell cycle progression [36]. Cyclin E, another member of the cyclin family, binds to G1 phase Cdk2 and is also required for the transition from G1 to S phase of the cell cycle. The Cyclin E/CDK2 complex tags p27^Kip1^ by phosphorylation, which promotes the degradation of p27^Kip1^, the expression of Cyclin A, and the progression of cell cycle into S phase [37]. Skp2 is an F-box protein belongs to the SCF (Skp1-Cullin 1-F-box protein) E3 ubiquitin ligase complex which regulates the S phase entry of cells by inducing the degradation of the cyclin-dependent kinase (Cdk) inhibitors p21^Cip1^, p27^Kip1^, p57, p130, Tob1, and FoxO1 [38–40]. Skp2 targets Cdk inhibitor p27^Kip1^ by phosphorylating p27^Kip1^ at T187 for ubiquitination and degradation [41–43]. Overexpression of AKT3 increased cyclin D1, cyclin E, and Skp2 but decreased p27^Kip1^ (Figure 6) which may explain how elevation of AKT3 promotes cell cycle progression in Figure 4.

Elevation of AKT3 protein expression can be caused by gene amplification, upregulation of mRNA transcription, increase of protein stability, and decrease of protein degradation. Our observation suggested that AKT3 mRNA is higher in prostate tumors as compared to normal prostate tissues, suggesting that upregulation of transcription can at least partially contribute to the increase of AKT3 protein expression. In future, we would like to determine if the stability and protein degradation of AKT3 is different between non-malignant vs. malignant prostate cells. Phosphorylation of threonine 308 on AKT is activated by PDK1 [44] while Phosphorylation of serine 473 on AKT is activated by mTOR kinase, its associated protein rector, and SIN1/MIP1 [45, 46]. We observed that phosphorylation of Thr308 and Ser473 on AKT both increased when we overexpressed AKT3 in PC-3, DU-145, CA-HPV-10, and LNCaP cells. However, we don't know whether elevation of AKT3 in prostate tumors directly contributes to the increase of phosphorylation of AKT in prostate tumors or not. Previous studies indicated that greater phosphorylation of AKT on serine 473 correlates to higher Gleason grades [47] and worse clinical outcome in prostate cancer [6]. Due to the limitation of clinical samples, we unfortunately can not determine the level of phosphor-AKT by IHC staining in our study. However, we believe that the elevation of AKT3 protein at least partially increases the phosphorylation of AKT on Ser473 and Thr308 in prostate tumors. Our observation suggested that overexpression of AKT3 in prostate cancer cell lines dramatically enhanced the phosphorylation of AKT on both Thr308 and Ser473, indicating that the activity of AKT3 increased significantly. TSC1 is also known as hamartin. TSC1 is a potent negative regulator of mTORC1 signaling and is an epithelial tumor suppressor preventing spontaneous PCa development in murine model [48]. TSC1-deficient mice developed prostatic intraepithelial neoplasia (PIN) and later prostate carcinoma [48]. TSC2 is also called Tuberin. Mutations in TSC2 gene lead to tuberous sclerosis. Gene product of TSC2 is believed to be a tumor suppressor and is able to stimulate specific GTPases [49]. TSC2 functions with a multi-protein complex known as the TSC complex which consists of the core proteins TSC2, TSC1, and TBC1D7 [49]. Constitutively active PI3K or active AKT, including AKT1 and AKT2, induces phosphorylation of TSC2 [50]. TSC2 is a direct physiological substrate of AKT. Phosphorylation of TSC2 by PI3K/AKT is a major mechanism controlling TSC1-TSC2 function [50]. AKT associates with TSC1-TSC2 complexes, promoting phosphorylation of TSC2, and increases degradation of TSC1-TSC2 complexes [50]. AKT also inhibits TSC2-mediated degradation of p27^Kip1^, thereby promoting Cdk2 activity and cellular proliferation [50]. Mutations in the PI3K/PTEN/TSC2 pathway contribute to mammalian target of rapamycin (mTOR) activity and increase translation under hypoxic conditions [51]. Although elevated mTOR activity and protein synthesis does not translate into enhanced cell proliferation rates, lack of TSC2 results in a survival advantage for cells being exposed to hypoxia [51]. Our study revealed that elevation of AKT3 protein can lessen the protein abundance of TSC1 and TSC2, which in turn stimulates the proliferation of PCa cells. Activation of B-Raf initiates the development of PCa in transgenic mice model [52]. AKT stimulates phosphorylation of B-Raf Ser445 and ERK1/2 activation in PCa cells in response to androgen depletion [53]. Our observation indicated that overexpression of AKT3 augmented protein abundance of B-Raf, which promotes the proliferation of prostate cancer cells. Down-regulation of TSC1 and TSC2 as well as up-regulation of B-Raf caused by elevation of AKT3 may therefore contribute to the increase of cellular proliferation and provide growth advantage for prostate cancer cells.

Our observation showed that mRNA and protein expression of AKT3 was higher in primary prostate tumors and that elevation of AKT3 promotes growth of prostate cancer cells. We believe that treatment with AKT3 inhibitor, such as Isoselenocyanates [54], may suppress the growth of primary prostate tumors. We also demonstrated that elevation of AKT3 increased resistance of PCa cells to B-Raf inhibitor treatment, while knockdown of AKT3 increased sensitivity of PCa cells to B-Raf inhibitor (Figure 10). This finding suggested the possibility that combination of AKT3 inhibitor with B-Raf inhibitor may be a potential therapy for advanced prostate tumor. In conclusion, our observations suggested that elevation of AKT3 promotes the proliferation of prostate cancers cells through regulation of Akt, B-Raf, & TSC1/TSC2.

## MATERIALS AND METHODS

### Cell culture

PC-3, DU-145, LNCaP, and CA-HPV-10 cells were purchased from Bioresource Collection and Research Center (Hsinchu city, Taiwan). PC-3, LNCaP, and DU-145 cells were maintained in DMEM (Gibco/Invitrogen, Carlsbad, CA, U.S.A.) supplemented with 10% fetal bovine serum (FBS; Atlas Biologicals, Fort Collins, CO, U.S.A.), penicillin (100 U/ml), and streptomycin (100 μg/ml) as previously described [23]. CA-HPV-10 cells were cultured in keratinocyte-serum free medium (Gibco) with 5 ng/ml human recombinant epidermal growth factor and 50 μg/ml bovine pituitary extract.

### Cell proliferation assay

Relative cell number was analyzed by measuring DNA content of cell lysates with the fluorescent dye Hoechst 33258 (Sigma, St. Louis, MO) as described previously [24–26]. For all proliferation assays, we included at least 24 repeats for each condition.

### Overexpression and siRNA knockdown of AKT3

For overexpression of AKT3, cells were seeded at 4.75 × 10^5^ cells/well in a 6-well plate in DMEM containing 10% FBS. 18–24 h after plating, cells were transfected with pCMV6-Entry Vector or AKT3 plasmid (OriGene, Rockville, MD, U.S.A.). PolyJet™ *in vitro* DNA transfection reagent was used (SigmaGen Laboratories, Rockville, MD). For siRNA knockdown of AKT3, both human AKT3 antisense and randomly scrambled sequence control were purchased from Thermo (Waltham, Massachusetts, U.S.A.). The transfection procedure was performed using lipofectamine RNAiMAX (Invitrogen, Carlsbad, CA, U.S.A.) according to the manufacturer's recommended protocol and 25 nM RNA were used for both scramble and AKT3 knockdown.

### Western blotting analysis

Western blots were performed as previously described [24–27]. Protein extracts were lysed in mammalian cell lysis buffer. Protein concentration was determined with the Bradford reagent (Bio-Rad Laboratories, Hercules, CA, U.S.A.) using a bovine serum albumin standard. Proteins were separated on 8–10% SDS-PAGE gels. Protein expression was determined by Western blotting using following antibodies: phospho-AKT S473, phospho-AKT T308, β-catenin, GSK3α, GSK3β, phospho-GSK-3α S21, phospho-GSK-3β S9, cyclin D1, cyclin E, mTOR, and total AKT antibodies were from Cell Signaling (Danvers, MA, U.S.A.); c-Myc, TSC1, TSC2, B-Raf, and phospho-p70S6K T421/S424 antibodies were from Epitomics (Burlingame, CA, U.S.A.); p21^Cip1^, p27^Kip1^, and Skp2 antibodies were from Santa Cruz (Santa Cruz, CA, U.S.A.); AKT3, phospho-mTOR S2448 and p70S6K antibodies were purchased from Millipore (Billerica, MA, U.S.A.); β-actin antibody was from Novus (Littleton, CO, U.S.A.);α-tubulin antibody was from Sigma (St. Louis, MO, U.S.A.). Anti-rabbit and anti-mouse IgG secondary antibodies were from Santa Cruz (Santa Cruz, CA, U.S.A.). α-tubulin and β-actin were used as loading control.

### Quantitative real-time pcr

The AKT3 mRNA level of human prostate tissue and different stages of prostate cancer was determined on TissueScan Prostate Tissue qPCR Array HPRT501~503 (OriGene Technologies, Rockville MD) according to the manufacturers' instruction with Maxima SYBR Green/ROX qPCR Master Mix (2x) (Fermentas, Glen Burnie, MA, USA) and analyzed by Applied Biosystems 7500 Real-Time PCR system (Thermo Scientific Waltham, Massachusetts, USA). The mRNA expression level was normalized to β-actin. The primer for AKT3 was designed using software on http://frodo.wi.mit.edu/primer3/, the forward sequence is GGAGTCATCATGAGCGATGTT, and the reverse sequence is AAGGAAGTATCTTGGCCTCCA. The mRNA level of AKT3, TSC1, TSC2, and BRAF in different prostate cancer cell lines was determined using qPCR kit Maxima SYBR Green/ROX qPCR Master Mix (2X) (cat. K0222) (Thermo Scientific Waltham, Massachusetts, U.S.A.). Applied Biosystems 7500 Real-Time PCR system was used with the Two-step cycling protocol for target cDNA product amplification (http://www.appliedbiosystems.com.tw/). The primer sequences for TSC1, TSC2, and BRAF are shown as following: TSC1: TTACCTGGAAACCAGCTCTCA (forward), GCTTTGCCCACATATTCGTTA (reverse); TSC2: AGAACTGAGCATGGAATGTGG (forward), CCTGCTCTTCAAATTTCTTGG (reverse); BRAF: TTCCTGATGGGCAGATTACAG (forward), CATGCCACTTTCCCTTGTAGA (reverse).

### Online dataset analysis

Level of AKT3 mRNA in dataset on PubMed GEO Profile from GDS3289 [28] (12 normal prostate stromal tissue, 18 normal prostate epithelial tissue, 32 primary prostate tumors, 20 metastatic prostate tumors; Reporter: GPL2013, Hs6–9-12–3 (ID_REF), GDS3289, IMAGE:592498) and in dataset from Oncomine of Grasso Prostate dataset (28 normal prostate gland tissues and 59 primary prostate tumors, reporter: A_23_P160354; Agilent Human Genome 44K) [29] was analyzed and plotted with SigmaPlot. *p* value smaller than 0.05 was considered statistically significant.

### Patients and specimens

The sample collection, research design, and consent procedure were approved by the Institutional Review Board of Taichung Veterans General Hospital. Written informed consent was obtained from every participant involved in this study. A series of 38 patients with prostate cancer having transurethral resection of the prostate (TURP) at Taichung Veterans General Hospital, Taichung, Taiwan, were enrolled from 1999 January to 2001 December. Further examination of biopsy specimens or resected tissue confirmed the diagnosis of prostate cancer. Paraffin embedded tissues were composed of cancerous parts and surrounding noncancerous prostate tissue were studied. These patients were followed for an average of 44.0 ± 19.6 months. The pathologic and clinical features of these patients including primary tumor, regional lymph node, Gleason's score, and stage were analyzed.

### Immunohistochemical analysis

Immunohistochemistry staining was performed on paraffin-embedded sections with an automatic immunostaining device and ultraView detection kit (Ventana XT Medical Systems, Tucson, AZ). Antigen retrieval was performed automatically by the device according to the manufacturers' instruction. The amount of AKT3 protein in tissue sections was analyzed using an AKT3 antibody [9B2] (ab84985) (1:200; Abcam, Cambridge, MA). Lung bronchial epithelium was stained as positive tissue controls, while prostate was used for negative reagent control. The intensity of immunostaining was scored semiquantitatively using a Quick score (Q-score) method based on intensity and heterogeneity. Briefly, the staining intensity was scored as 0 (negative), 1 (weak), 2 (moderate), or 3 (strong). For heterogeneity staining, the proportion of tumor cells stained for AKT3 was scored as 0, 1 (1%–25%), 2 (26%–50%), 3 (51%-75%), or 4 (76%-100%). The Q-score of a given tissue sample is the sum of the intensity and heterogeneity scores and ranges from 0 to 7. Those rare cases with less than 5% weakly stained tissue were considered negative. Scoring of each sample was made independently and blindly by 2 pathologists (Drs Jan and Wang). A change of Q-score between different groups of tumors was considered statistically significant if the *p* value was smaller than 0.05.

### Flow cytometric analysis

LNCaP cells with control plasmid or AKT3 overexpression were seeded at the density of 5 × 10^5^ cells in 6-cm dishes. After 4 days of culture in the presence of various concentrations of reagents, cells were collected and fixed in 70% ethanol/30% PBS overnight at −20°C. Fixed cells were washed with PBS, treated with 0.1 mg/mL RNase A in PBS for 30 minutes, and then suspended in 50 μg/mL propidium iodide in PBS. Cell cycle profiles and distributions were determined by flow cytometric analysis of 2 × 10^4^ cells using a BD Facscan flow cytometer (BD Biosciences, San Jose, CA). Cell cycle distribution was analyzed with build-in software of BD Facscan flow cytometer as previously [24–27]

### Gene correlation on-line data analysis

Correlation between AKT3 and BRAF genes was determined in Tomlins Prostate dataset (AKT3/IMAGE:360838; BRAF/IMAGE:417403; *R* = 0.61, *n* = 29) [28] and Yu Prostate dataset (AKT3 probe: 40781_at; BRAF probe: 40306_at; *R* = 0.31, *n* = 64) [30].

### Xenografts in athymic mice

Experiments involving mice were approved by National Health Research Institutes Institutional Animal Care and Use Committee. The study was carried out in strict accordance with the recommendations in the Guide for the Care and Use of Laboratory Animals of the National Institutes of Health. Male Balb/c nu/nu mice purchased from National Laboratory Animal Center (Taipei city, Taiwan) at age 6–8 weeks of age were injected subcutaneously in both flanks with 5 × 10^5^ PC-3 cells overexpressing either control vector or AKT3. Cells were suspended in 0.5 ml of Matrigel (BD Bioscience, Franklin Lakes, NJ, USA) and were injected subcutaneously into athymic mice to form tumors. After 2 weeks, the average tumor volume exceeded 100 mm^3^. The control group contained 7 mice and 11 tumors, while the AKT3 overexpression group contained 7 mice and 10 tumors. Tumor volume and body weight of mice carrying PC-3 xenografts was measured weekly using calipers and volume was calculated using the formula volume = length × width × height × 0.52 [31–34].

### Data analysis

Data are presented as the mean +/− SD of at least three experiments or are representative of experiments repeated at least three times. Student's *t* test (two-tailed, unpaired) was used to evaluate the statistical significance of results from proliferation assay experiments.
